# Supplementary material for: Splice-Junction-Based Mapping of Alternative Isoforms in the Human Proteome
Source: Cell Rep. Author manuscript; Available in PMC 2020 Jan 15. (PMC6961840; doi:10.1016/j.celrep.2019.11.026)
Supplement: 2 [file NIHMS1546469-supplement-2.zip › DF1/PXD006675/TricuspidValve/TricuspidValve_6_CCS_GSGQLRVQDVEVHLEDQMVLVHTTLPSQEVQALLEGTGR.pdf]

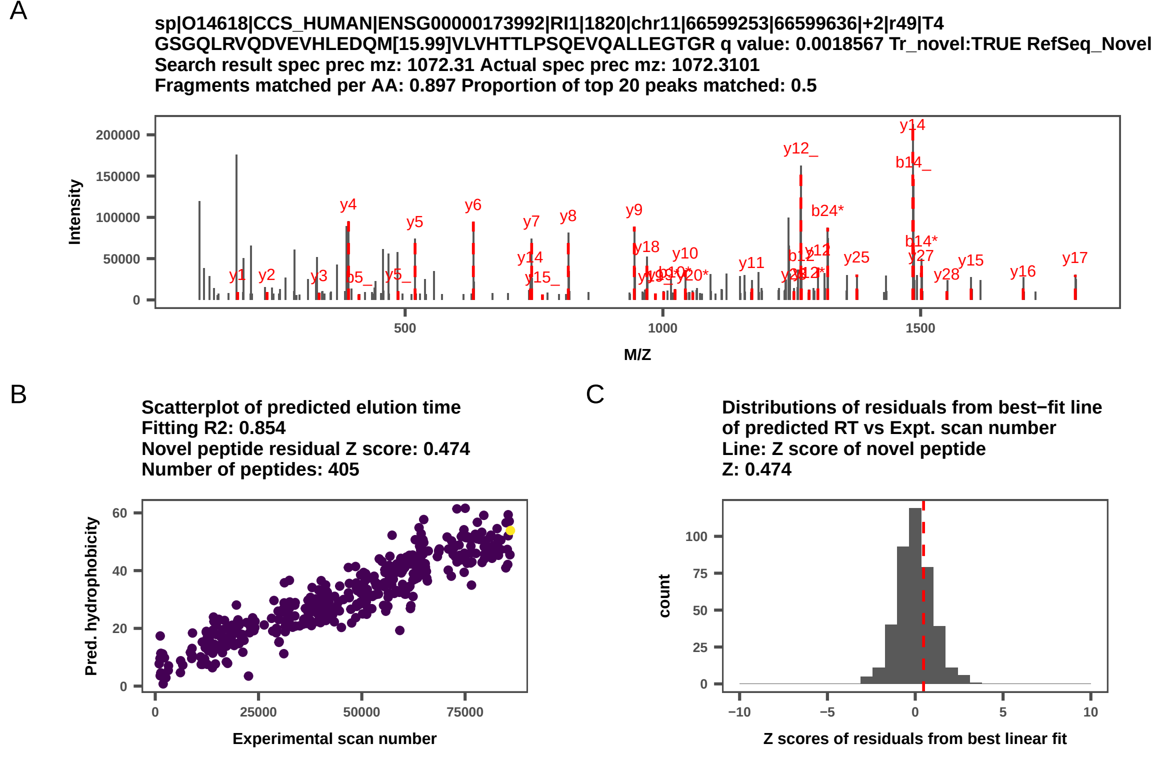
A
sp|O14618|CCS_HUMAN|ENSG00000173992|RI1|1820|chr11|66599253|66599636|+2|r49|T4
GSGQLRVQDVEVHLEDQM[15.99]VLVHTTLPSQEVQALLEGTGR q value: 0.0018567 Tr_novel:TRUE RefSeq_Novel
Search result spec prec mz: 1072.31 Actual spec prec mz: 1072.3101
Fragments matched per AA: 0.897 Proportion of top 20 peaks matched: 0.5
Intensity
200000
150000
100000
50000
0
500
1000
1500
M/Z
y1
y2
y3
y4
b5_
y5
y5_
y6
y7
y14
y15_
y8
y9
y18
y10
y11
y12_
b12
y12
b24*
y25
y14
b14_
b14*
y27
y28
y15
y16
y17
B
Scatterplot of predicted elution time
Fitting R2: 0.854
Novel peptide residual Z score: 0.474
Number of peptides: 405
Pred. hydrophobicity
60
40
20
0
0
25000
50000
75000
Experimental scan number
C
Distributions of residuals from best−fit line
of predicted RT vs Expt. scan number
Line: Z score of novel peptide
Z: 0.474
count
100
75
50
25
0
−10
−5
0
5
10
Z scores of residuals from best linear fit
